# Transcriptome Analysis of Core Dinoflagellates Reveals a Universal Bias towards “GC” Rich Codons

**DOI:** 10.3390/md15050125

**Published:** 2017-04-27

**Authors:** Ernest Williams, Allen Place, Tsvetan Bachvaroff

**Affiliations:** Institute of Marine and Environmental Technology, University of Maryland Center for Environmental Science, 701 East Pratt St., Baltimore, MD 21202, USA; williamse@umces.edu (E.W.); place@umces.edu (A.P.)

**Keywords:** dinoflagellate, toxin, codon bias, gene expression

## Abstract

Although dinoflagellates are a potential source of pharmaceuticals and natural products, the mechanisms for regulating and producing these compounds are largely unknown because of extensive post-transcriptional control of gene expression. One well-documented mechanism for controlling gene expression during translation is codon bias, whereby specific codons slow or even terminate protein synthesis. Approximately 10,000 annotatable genes from fifteen “core” dinoflagellate transcriptomes along a range of overall guanine and cytosine (GC) content were used for codonW analysis to determine the relative synonymous codon usage (RSCU) and the GC content at each codon position. GC bias in the analyzed dataset and at the third codon position varied from 51% and 54% to 66% and 88%, respectively. Codons poor in GC were observed to be universally absent, but bias was most pronounced for codons ending in uracil followed by adenine (UA). GC bias at the third codon position was able to explain low abundance codons as well as the low effective number of codons. Thus, we propose that a bias towards codons rich in GC bases is a universal feature of core dinoflagellates, possibly relating to their unique chromosome structure, and not likely a major mechanism for controlling gene expression.

## 1. Introduction

Along with several species of cyanobacteria, dinoflagellates have become quite infamous as the causative agents of harmful algal blooms [1]. Aquatic vertebrates and humans are adversely affected by these blooms directly and indirectly as the potent toxins produced by dinoflagellates, including brevetoxin, karlotoxin, ciguatoxin, and palytoxin work their way into the surrounding environment, often entering the food web through filter feeders [2]. Several pathologies have been described following exposure to these toxins including paralytic shellfish poisoning, neurotoxic shellfish poisoning, amnesic shellfish poisoning, diarrheic shellfish poisoning, and ciguatera fish poisoning. The production of these toxins is often for reasons unknown, and their synthesis is even less well understood, but, as with many natural products in the aquatic environment, there is a chance for discovery of novel drugs or valuable compounds. 

Further investigation into the protein complexes that make these toxins and their regulation has been extremely difficult, partly because the toxins themselves are often extremely complex compounds [3,4,5], but also because most gene expression in dinoflagellates is regulated post-transcriptionally [6,7,8]. Techniques such as quantitative reverse-transcription polymerase chain reaction (RT-PCR), microarrays, and transcriptome profiling are often inappropriate and potentially misleading for correlating changes in gene expression to phenotypes or environmental stresses. Current proteomics technologies can give some information, but the number of proteins involved in synthesizing complex molecules limits the usefulness of proteomics for studying toxin production. This hampers efforts to mine dinoflagellate species for potential pharmaceuticals or as a production system for valuable pigments or fatty acids like docosahexaenoic acid (DHA). The intractability of this unique and economically relevant group of algae will likely continue until more insight into their mechanisms of gene regulation can be gained.

There are several described methods that can regulate the expression of proteins post-transcriptionally including micro-RNAs [9], RNA maturation [10] and decay [11], and codon bias [12] which can slow or terminate translation of messages if any of the complimentary tRNAs are in low abundance. A suite of micro-RNAs and many of the corresponding open reading frames have been putatively annotated in *Symbiodinium microadriaticum* and in *Alexandrium catanella* using a transcriptome profiling method which selects for RNAs that have been processed by dicer, a necessary step in micro-RNA maturation [13,14]. Many more micro-RNAs are likely to be described in dinoflagellates as the databases containing annotated micro-RNAs increase, and the ways in which micro-RNAs can alter gene expression can be surprisingly dynamic. This is unlikely to be a means of regulating gene expression on a global scale, however, since it would require a complementary micro-RNA for all mRNAs. The demonstration of unusually long RNA half lives in *Karenia brevis* [15] means the micro-RNAs would need to prevent translation as well as stabilize their respective mRNA. Long RNA half lives also means that RNA abundance alone is unlikely to affect protein expression and that regulation is occurring at or during translation. 

Codon bias is an attractive method for global control of gene expression, likely along side other methods, because tRNA availability simultaneously affects all mRNAs being translated. The ability for codon bias to affect the speed of translation as well as to terminate translation prematurely has been demonstrated in many model species [16]. Transcripts whose expression is under the influence of codon bias are often evidenced by rare codons within the open reading frame with a different guanine cytosine (GC) content than the most commonly used codon for its respective amino acid [17]. These subpopulations of open reading frames can be differentiated based on codon frequency, which can be correlated with protein abundance [18,19]. Relative codon frequency is framed within the context of amino acid frequency and conservation as well as overall genome nucleotide composition. Additionally, with 64 codons and 20 amino acids, the number of codons for each amino acid ranges from one to six, with the second and first codon positions having the most specificity and third codon position the most flexibility for synonymous substitution [20].

For this study, whole transcriptomes were selected from fifteen dinoflagellate taxa across a range of overall proportion of GC content from 51% GC to 66% GC. The relative synonymous codon usage (RSCU) was calculated using the software codonW for a subset of annotated genes from each transcriptome, and comparisons were made across all species as well as for each sequence within each species looking for populations of RNA sequences with the hallmarks of codon bias [21]. Gene selection and species comparisons were anchored methodologically using *Amphidinium carterae* because it is the most basal photosynthetic core dinoflagellate, a toxin producer, and has a well assembled transcriptome (Genbank accession #SRX722011) [22,23]. Because these analyses are based on codon abundance it is critical that protein coding sequences in the proper reading frame are used. The results of this study reveal a lack of codon bias within specific groups of transcripts but rather a universal bias against uracil adenine (UA) dinucleotides encompassed by two of the three stop codons and all UA ending codons across all core dinoflagellate species. Codon bias within each species was defined by that species’ overall GC content and the concomitant change in the effective number of codons. This ultimately results in a streamlining of codon use with increasing GC content that is unlike what has been demonstrated in model organisms. GC content in the context of dinoflagellate evolution and a possible link to the dinokaryon are discussed.

## 2. Results

### 2.1. GC Content

#### 2.1.1. Total GC Content

**Figure 1:**
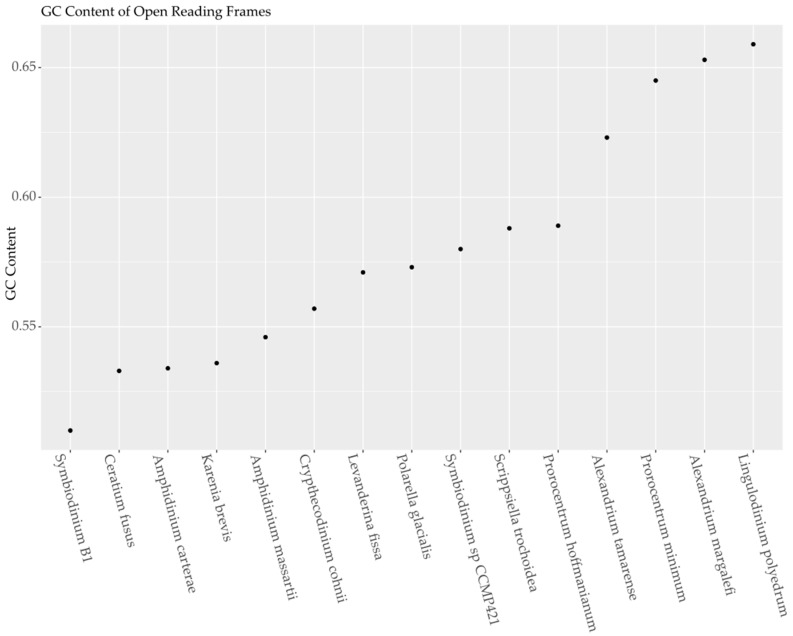
A guanine cytosine (GC) content versus species plot for the fifteen dinoflagellate transcriptomes used in this study. Species names for the corresponding transcriptome are given on the *X*-axis with strain numbers when applicable. The *Y*-axis shows the fraction of the overall nucleotide count of guanine and cytosine base pairs.

The total GC content of each transcriptome used in this study varied from 51% to 68% GC and were chosen from available transcriptomes to cover the full range of GC content by increments of approximately 2%. Of 69,356 total sequences from *A. carterae*, 12,578 putative coding regions had basic local alignment search tool (BLAST) hits with an e-value of less than 1 × e^−10^ to the translated reference sequence database at the national center for biotechnology information (NCBI). After recovering sequences from the remaining transcriptomes with BLAST matches to the reference open reading frames from *A. carterae* using an e-value cutoff of 1 × e^−10^, the GC content across all tested transcriptomes varied from 51.0% to 65.9% with a slight reduction in GC content for most species with the exception of *K. brevis* (Figure 1). When the regions corresponding to these hits were extracted, there were approximately 1.8 million codons and 88 stop codons in the *A. carterae* dataset. Across the remaining species the number of open reading frames varied from 1862 to 9848, with an average of 7500. The total number of codons per species was 0.6–3.2 million (average of 2.3 million). Stop codons were present in only a very small fraction of sequences (0.6% on average). Summary statistics from the codonW output for the fifteen species are presented in Table 1. The analyzed dataset was subsampled from the full transcriptomes to reduce contaminants while still covering a wide range of GC content with an ample set of open reading frames.

#### 2.1.2. GC Content by Codon Position

**Figure 2:**
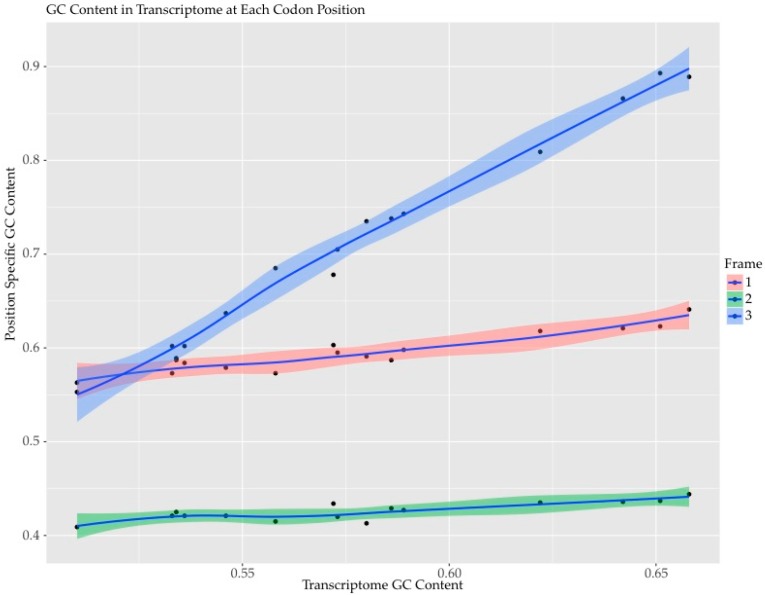
Plot of guanine cytosine (GC) content at each codon position versus the overall GC content of the transcriptome for each of the fifteen species. The *X*-axis shows the GC content of the transcriptome as a fraction of the total number of nucleotides. The *Y*-axis shows the GC content of the codon positions as a fraction of the total number of nucleotides with codon positions one, two and three colored pink, green, and blue respectively.

The first, second and third codon positions showed different patterns when compared across these species (Figure 2). The first and second positions had a much lower total range of difference than the third position. The values for third codon positions and for synonymous GC bias in the third position (GC3s) started at slightly biased values (55%) and approached a maximum of 90% GC content. Position two was slightly AT biased (40% to 45% GC), but varied only slightly across species. Position one was slightly GC biased with a broader range than position two (56% to 63% GC). The universal bias at codon position one and two above and below 50%, respectively, is likely influenced by the absence of stop codons (UAA, UGA, and UAG) in the analyzed dataset and rare amino acids such as tryptophan (UGG) and does not correlate with increasing GC content in the transcriptome.

### 2.2. Codon Bias and Effective Codon Number

**Figure 3:**
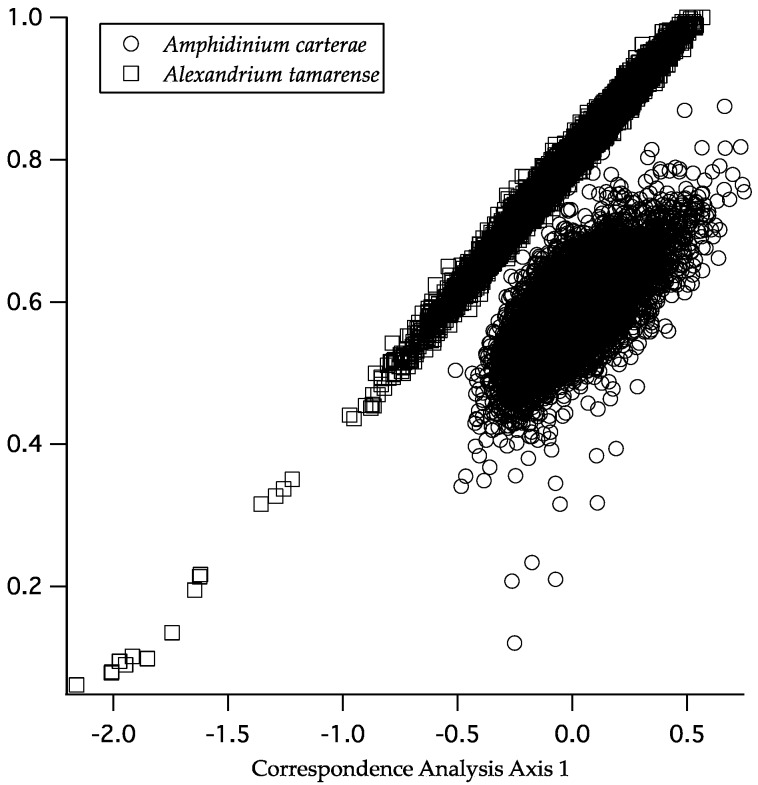
Correspondence analysis output from codonW for *Amphidinium carterae* and *Alexandrium tamarense* The *X*-axis is based on the eigen values from the principal component analyses based on codon bias and the *Y*-axis reflects synonymous third position guanine cytosine (GC) content.

Two species, *A. carterae* and *Alexandrium tamarense*, were selected for correspondence analysis with codonW. These species are found on the extremes of the GC content axis, expressed either as total GC or GC3s as shown in Table 1. The correspondence analysis in codonW selects genes that use relatively few codons per sequence and genes that use a more diverse set of codons. These extremes can be described by the measure of the effective number of codons (ENc). For *A. carterae*, there were 50 to 56 codons per sequence and 25 to 60 for *A. tamarense*. Considering the sequences that use fewer codons as having a form of codon bias, the correspondence analysis then ordinates each sequence along an axis (Figure 3). The variation in the data explained by the first axis in *A. carterae* was relatively small, as seen by the tight clustering on this axis. In the case of *A. tamarense*, synonymous GC content (GC3s) was an excellent predictor of the position of each gene by correspondence analysis. Thus, increasing GC content in the transcriptome increased the correlation between codon bias correspondence and GC3swithout subpopulations in either species.

**Figure 4:**
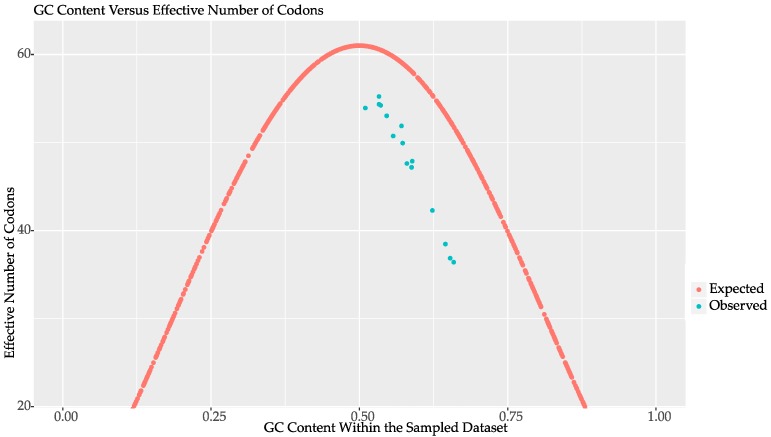
The effective numbers of codons are shown relative to the guanine cytosine (GC) content of the transcriptome for each of the fifteen species. The *X*-axis is the GC content as a fraction of the total number of bases while the *Y*-axis is the total number of effective codons excluding stop codons. The expected number of codons shown in red is the maximum number of codons predicted for a randomly generated dataset of one thousand points while the observed values in blue are the average effective number of codons predicted by codonW given the tabulated relative synonymous codon usage for each species.

**Figure 5:**
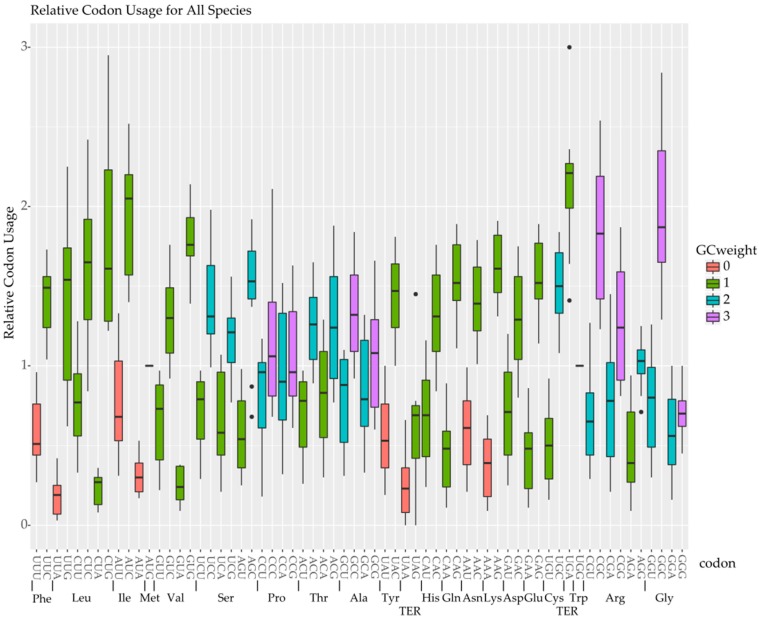
The relative synonymous codon usage for each codon is shown as a boxplot aggregating the data from all fifteen species. Boxplots are colored according to the “guanine cytosine (GC) weight” of each codon, i.e., the number of G or C bases occurring in the codon ranging from zero to four. Codons are arranged according to their respective amino acid given underneath in brackets.

The global plot of ENc for each species mimicked the overall GC content plots, in that GC-biased species used fewer codons per sequence and were skewed towards the GC biased end of the plot (Figure 4). However, across all species including the least biased, there was an offset from the maximum number of available codons at a given GC content of about four codons per transcript. Comparing the relative synonymous codon use (RSCU) patterns across all 15 species and 59 variable codons (ATG and TGG are single codon amino acids, and TGA, TAA, and TAG are excluded) revealed that codons ending in UA, AU, and the three non-terminator AA ending codons were significantly less frequent on average across all the species (Figure 5). This codon bias is obviously correlated with GC content as shown in the GC biased datasets, but was also present in the less biased datasets for these four codons. For example, selecting out the four most neutral datasets, *A. carterae*, *Symbiodinium* sp. B1, *Ceratium fusus*, and *Karenia brevis*, showed the four codons ending in UA were the least used, all with RSCU values less than 0.4. These four UA ending codons encode three amino acids: leucine, isoleucine, and valine. Leucine has six codons so the RSCU values will sum to six. The codons UUA (0.30) and CUA (0.19) were less frequent, and UUG (1.83) and CUG (1.39) were more frequent while the remaining two codons had values slightly above one. Isoleucine has three codons, of which AUA (0.38) was least frequent while AUC (1.48) and AUU (1.15) were more frequent. Valine has four codons and GUA (0.37) was infrequent while GUG (1.73) was most frequent and the other two codons were near one. Thus, in the most GC neutral datasets for the four amino acids with codons ending in UA, three of them preferred UG ending cognates.

**Figure 6:**
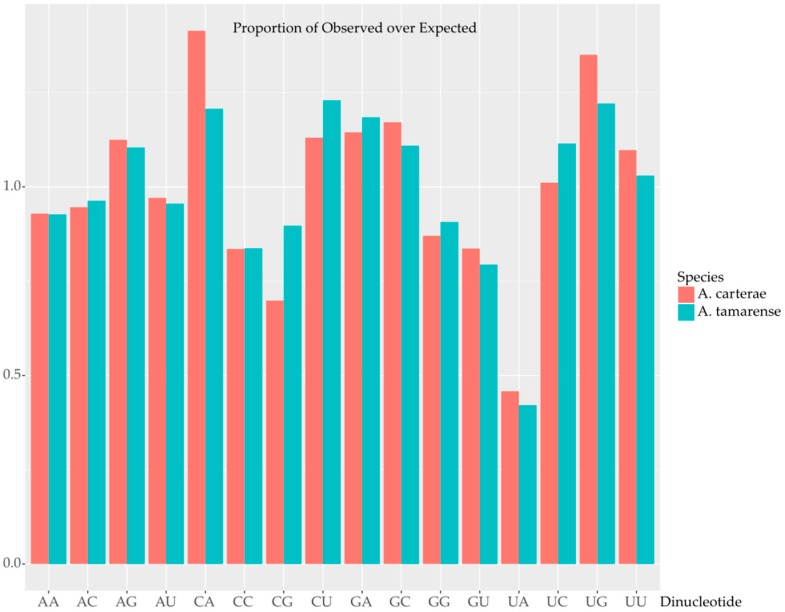
Dinucleotide frequencies are given as the ratio of observed over expected frequencies. Expected frequencies were calculated from the mononucleotide frequencies. The guanine cytosine (GC) neutral species *Amphidinium carterae* is shown in red while the relatively GC rich species *Alexandrium tamarense* is shown in cyan. All sixteen possible dinucleotide combinations are shown on the *X*-axis while the relative ratios are on the *Y*-axis.

This bias against UA ending codons can also be seen in the dinucleotide analysis where UA is observed in a much lower frequency than expected based on mononucleotide frequencies (Figure 6). In addition, the ratio of observed and expected frequencies is strikingly similar between the GC neutral and GC rich test species irrespective of GC content. There are some subtle deviations from a ratio of 1.00 for other dinucleotides likely due to relative codon preference that is species specific.

**Figure 7:**
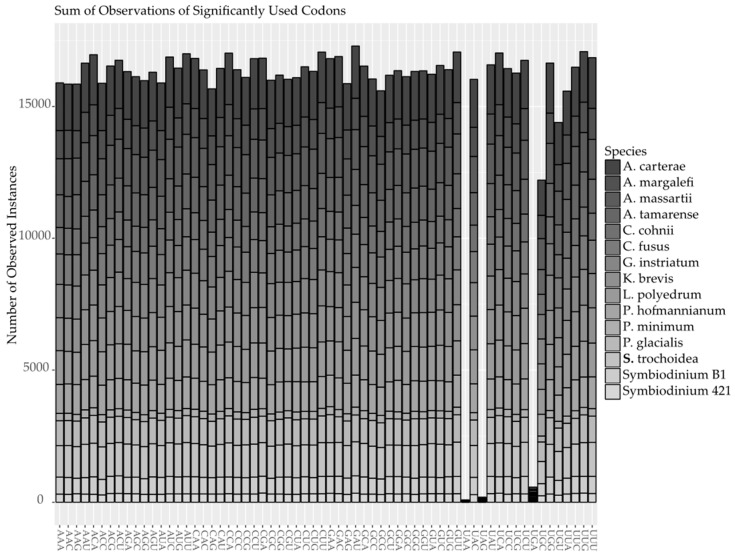
The number of times each codon occurred at a significantly higher than average frequency is shown for each codon. Columns are stacked and color coded for each species used in the analysis according to the legend. The *X*-axis shows each of the possible 64 codons, including the stop codons TGA, TAA and TAG. The *Y*-axis is the sum of observations across all species.

A bias against AU rich codons can be seen across species, but within sequence bias was observed across all codons equally. The means and standard deviations of observed codon frequencies within a sequence were similar between species ranging from 0.0028 and 0.0040 to 0.0509 and 0.0234, respectively, excluding stop codons. Recording codons observed within a sequence at a frequency higher than the mean plus a standard deviation resulted in approximately 915 thousand positives out of 7 million observations or 13%. This was effectively random across codons with approximately 15 thousand occurrences of high frequency for each codon, excluding stop codons (Figure 7). The exception was cysteine, which had the lowest number of observed within sequence codon bias for its two codons. This would indicate that in sequences with multiple cysteines, identical codons would be encountered slightly more frequently than combinations of synonymous codons but that all other codons would be found at a relatively equal frequency.

## 3. Discussion

Codon bias is an important mechanism for translational control of gene expression that exploits the error-prevention mechanisms of the ribosome [24,25]. The global nature of this phenomenon to control gene expression gives codon bias the potential to explain the enigmatic reliance on post-transcriptional control of gene expression in dinoflagellates. Toxins made by dinoflagellates can be large complex structures and are likely produced by polyketide synthases and/or non-ribosomal protein synthases that are subsequently modified to their final form [26,27,28]. Toxin production, release, and modification can be correlated to a host of genotypic and environmental factors, implying a complex regulatory network. If codon bias is playing a role in the global control of dinoflagellate gene expression, populations of genes could be observed with different codon preferences ultimately resulting in more or less efficient translation of those messages and changing the amount of protein made. This could then in turn be exploited to begin manipulating pathways for toxin, carotenoid, and fatty acid synthesis and harvest these valuable natural products. Codon bias, in itself, reflects a compromise in information content between the 64 possible codons and the 20 amino acids plus stop codons. The number of codons per amino acid varies from one to six, as does the GC content of each codon across each amino acid. All glycine codons start with GG, while all lysine codons begin with AA, and the relative frequency of these amino acids varies as well. Some amino acids are readily substituted for each other while others are rare or less easily substituted. Differences in codon use are often specified at the most flexible third codon position where changes result in the same amino acid translation. Usually, there is a correlation with GC content of the genome and the GC third position bias of optimal codons [20]. Codon analysis has been a powerful tool in developing gene expression systems and understanding the process of translation and gene expression.

Genomic GC content is consistently biased towards higher GC within “core” dinoflagellates in contrast to sequenced syndinian dinoflagellates which are often parasitic and AT rich [29], but is otherwise not strongly constrained. Varying genomic GC content across species was reflected in bulk transcripts, coding regions, and codon positions in this study and has been shown to vary more than 5% among strains of the same species [30]. The transcript data closely reflect global genome GC content, but there is almost always a slight reduction in GC content in the coding region indicating that non-coding regions are more GC rich, although the effect was often subtle. A caveat is that the sequences processed for codon analysis are those with good BLAST hits and neither represent all of the reading frames in the transcriptome nor the complete reading frame. The BLAST approach does bypass potential artifacts such as frameshifts, alternative splicing, and gene fragments. Overall, the coding regions likely represent a good cross section of the coding potential. In species that had bulk GC content greater than 65% the third position was dramatically biased, with GC3s approaching 90%. However, even amongst less strongly biased species, GC3s was consistently higher than the other positions (Figure 2). This led to a focus on two exemplars representing the two extremes of GC bias for correspondence analysis. 

The correspondence analysis for the nearly GC neutral species *Amphidinium carterae* and the GC rich species *Alexanrium tamarense*, showed two distinct patterns (Figure 3). The codon bias in *A. tamarense* is almost perfectly correlated with GC bias at third positions, and is more streamlined with fewer potential outliers than that of *A. carterae*. Thus for the GC biased species codon preference simply reflects GC content at synonymous sites. The *A. carterae* transcripts show a narrow range of GC3s bias that is not strongly correlated with the correspondence analysis axis, but the transcripts from *A. carterae* are also relatively closely clustered on that axis in comparison to *A. tamarense*. This close clustering is because in *A. carterae* there was little difference in the ENc across transcripts. While the location of each transcript on the correspondence analysis axis cannot be compared between species, we can see an overall trend where an increase in GC content and the concomitant reduction in the effective number of codons causes a constraint on synonymous substitutions at the third codon position. There is also a marked lack of subpopulations where codon bias is differentially explained, which argues for a lack of codon bias that would differentially affect translation. Furthermore, members of the eIF4E gene family that are presumed to have different functional roles and thus different expression patterns [31] all occur within the core of the scatterplots arguing strongly against codon bias playing a role in regulating gene expression globally. A lack of transcripts that are differentially biased in their use of infrequent codons is in agreement with both the observation of long RNA half lives in dinoflagellates [15] and the observation that RNA turnover is much more rapid in RNAs with non-optimal codons [32]. It could be that the mechanisms behind post-transcriptional control in dinoflagellates have reduced the overall effectiveness of codon bias in affecting translation rates while simultaneously stabilizing mRNAs waiting to be translated. Expanding the analysis to the remaining species in the ENc plot summarized a general pattern across the core dinoflagellates (Figure 4). For species with GC bias, there are a relatively smaller numbers of codons per transcript, while for species that are less GC biased a larger number of codons are used, albeit with a consistent offset towards GC (on the *X*-axis) with approximately four codons less than the maximum (on the *Y*-axis). When comparing RSCU values within and across species this result was further confirmed. Thus GC bias in the genome and its ultimate impact on the effective number of codons is what appears to be shaping the results of the correspondence.

The deviation in the observed versus the expected effective codons can be explained by the four codons ending in UA, which are universally the four least commonly used codons in core dinoflagellates in the analysis of RSCU (Figure 5). This result was consistent when calculated on a per species basis, across all the species, and was also found when looking only at the most GC neutral taxa. This bias fits very well with the general pattern of at least some GC bias in dinoflagellates and no observations of species with an AT bias, at least within “core” dinoflagellates [29]. Interestingly, while these codons are universally shunned, the favored replacement codons vary across the species. The bias against AU ending codons is reflected on a dinucleotide level bias, as the AU dinucleotide is clearly less frequent than expected based on base composition and this dinucleotide is also contained in two of the three potential stop codons (Figure 6). Several predictions can then be made on this basis: tRNA complimentary to AU ending codons will be less frequent, that transcripts with more AU ending codons would be more slowly translated, and that transfection constructs should avoid these codons. 

**Figure 8:**
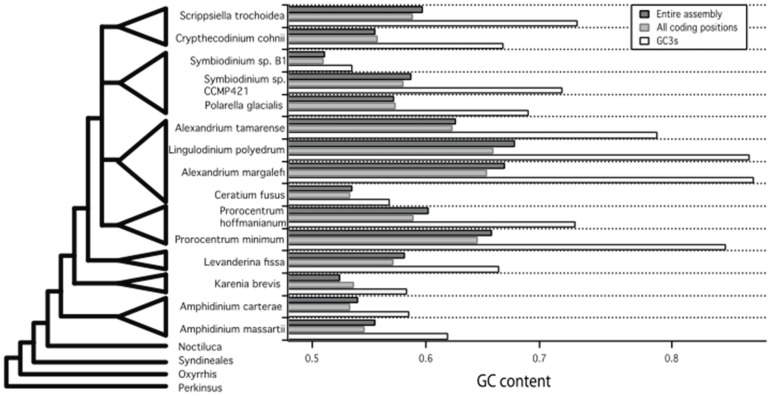
A phylogeny of dinoflagellates drawn using the branching order described in [33] with the guanine cytosine (GC) content for each of the fifteen species used in this study mapped onto the tree according to its own position or that of the most closely related species. GC content is shown on the *X*-axis as the relative proportion of GC bases pairs out of the total number of nucleotide base pairs in the transcriptome for each species plotted.

One important question is whether the codon bias and dinucleotide bias is a cause or consequence of the general GC bias trend? High GC content (>60%) has evolved multiple times during the evolution of this group, if a GC neutral ancestor is proposed. Mapping GC content onto the phylogeny from [33], we can see at least two independent evolutions of GC content above 60% (Figure 8). Indeed, within four genera (*Amphidinium*, *Symbiodinium*, *Prorocentrum*, and *Alexandrium*), a range in global, coding and GC3s values were seen. In terms of genomic nucleotide content, there are also differences within species. These results suggest flexibility in GC content across core dinoflagellates, albeit with strict limits against AT bias.

Thus, the discrimination against certain codons in all of the core dinoflagellate species used in this study may not give a complete picture of potential codon bias, requiring a more in depth look at codon use within each sequence for each species. By calculating the mean and standard deviation of the frequency of each codon within a sequence for each species it was possible to apply a simple metric for significantly increased use of a codon within a sequence by asking if it occurred at a frequency greater than the mean plus a standard deviation for that codon and species. The reverse was not possible since specific amino acids and therefore all the corresponding codons can be absent from a sequence resulting in false positives. We can see in Figure 6 that all codons, excluding stop codons, for all sequences within each species occur in a higher than expected frequency approximately the same number of times. Codons for cysteine are a possible exception with slightly lower observation than the remaining amino acid codons, but it does not appear that codon bias is preferential for one or more specific codons that would be employed as key regulators of translation efficiency. If there is a universal trend towards high GC content in the core dinoflagellates, selection pressure may not be acting on codons or the RNA environment at all but rather the DNA environment. Core dinoflagellates have a unique chromosome structure called the dinokaryon in which there is no evidence for nucleosomes as the structural protein and has been replaced by a major basic nuclear protein and a dinoflagellate viral nuclear protein [34]. There is also an abundance of divalent cations surrounding the chromosomes whose helices are in the rare Z-conformation rather than the ubiquitous B-conformation [35]. This results in a chromosome structure that is permanently condensed and birefringent with a crystalline appearance [36,37,38]. High GC content in the genome may serve to stabilize this chromosomal structure or differentiate regions of the chromosome in a manner that is not fully understood.

The initial hypothesis was that codon bias might play a major role in controlling gene expression in dinoflagellates, such that preferred codons would be directly linked to transcripts with codon bias. However, these results suggest only that four specific codons are very infrequently used in core dinoflagellates and do not reveal strong patterns of codon bias in specific transcripts. The lack of evidence for any codon bias that could act on translation may indicate different mechanisms for interactions between transcripts and tRNAs within the ribosome than what has been described in model organisms. Unfortunately, currently available dinoflagellate genomes are incomplete so an exhaustive list of tRNAs for each species is unavailable [39,40,41]. The tRNA genes that have been annotated in these genomes, however, as well as the genome of the closely related species *Perkinsus marinus* (Genbank accession GCA_000006405.1), are surprisingly depauperate of tRNAs for GC rich codons such as CGC (arginine), GCC (alanine), and UCC (serine) (data not shown). This is in contrast to the transcriptome based findings presented in this study and further research may uncover the biological mechanisms responsible for the apparent lack of codon bias in core dinoflagellates and may even lead to drug classes that could specifically target dinoflagellates and help mitigate harmful algal blooms. Unfortunately, regulation of gene expression in dinoflagellates remains a mystery and canonical mechanisms may have been lost during their evolution and replaced by other more cryptic devices. It may be that RNAs are sequestered and that the majority of regulation is acting just prior to or during translation initiation. Further investigations into RNA structure and RNA interacting partners will hopefully help to unlock the biotechnological potential of these unique organisms.

## 4. Materials and Methods

### 4.1. ORF Extraction

Illumina reads for each species were assembled using Trinity, as previously described [42]. The GC content of all sequences in each core dinoflagellate transcriptome assembly was calculated using a Perl script. A BLAST-guided approach was used to extract coding regions from the transcriptomes on a species by species basis. First, all assembled nucleotide sequences greater than 500 bases from *Amphidinium carterae* were used as queries against the NCBI/GenBank reference sequence protein database with an e-value less than 1 × e^−10^ [43]. The start and stop coordinates and reading frame of the query versus the top BLAST hit were then extracted and used to select only the nucleotides covered by the BLAST alignment and then output the results in the +1 reading frame using a Perl script. This starting dataset using length and rigorous BLAST score cutoffs was necessary to reduce the number of fragmented sequences and contaminants from organelles or bacteria in co-culture that were retained following poly-adenosine selection during transcriptome sequencing. This will result in a much smaller dataset, however relative to the total transcriptome with some removal of the ends of each open reading frame. A similar approach was used for the remaining dinoflagellates, but the reference sequence database was bypassed. The sequences from *A. carterae* collected above were translated into amino acids and used as queries against individual dinoflagellate transcriptomes using tBLASTn (amino acid query versus translated nucleotide database) with an e-value cut-off of 1 × e^−10^. The results were parsed as above to extract regions with a BLAST match and output the sequences in the +1 reading frame. The total number of stop codons was tabulated for each species as an estimator of the fraction of contaminating sequences based on the assumption of the “universal” genetic code. 

### 4.2. Relative Synonomous Codon Usage Analyses

The nucleotide sequences were used as input to codonW in several ways [21]. First the RSCU values were calculated across every sequence from each species (using the codonW “total” flag). Second, the relevant codon statistics were calculated for each sequence (using the “all_indices” flag) and averages calculated using excel. For selected species a correlation analysis was performed in codonW. Expected dinucleotide frequencies were calculated based on observed frequencies each nucleotide and multiplying them for each of the sixteen possible combinations. Mathematical manipulations and graphing were done using the R programming language version 3.3.2. RSCU values among codons and amino acids were generated using the ggplot package in R with the geom_boxplot function. Plots of GC content by position and effective number of codons were also made with the ggplot package using the geom_smooth and geom point functions, respectively. The simulated dataset for the effective number of codons plot was generated using the runif function with a range of 0 to 1 and 1000 iterations. A codon within a sequence was determined to be biased if it was present at a frequency higher than the mean frequency of that codon for each species plus one standard deviation. Codons with very low frequencies could not be quantified since in many proteins specific amino acids were absent resulting in a high count of zero frequencies.

## Figures and Tables

**Table 1:** Summary statistics from the output of codonW are shown. The left column gives the species name as well as the strain if known and the accession in NCBI’s Genbank for the transcriptome used. The codonW output from the subsampled dataset shown in the middle columns gives, from left to right, the number of putative protein coding sequences as “Genes”, the total number of codons, the number of stop codons annotated using standard protein translation, the codon bias index (CBI), frequency of optimal codons (Fop), effective number of codons (Nc), and the GC content at codon position three resulting in synonymous substitutions (GC3s). The species are vertically sorted from low to high GC content of their full transcriptomes.

Species Name	Genes	Codons	Stops	CBI	Fop	Nc	GC3s	Transcriptome GC Content
*Symbiodinium* sp. B1 DRX021274-5	4270	1,445,562	19	0.007	0.420	53.911	0.535	0.510
*Ceratium fusus* SRX554108-9	8133	2,611,156	47	0.047	0.443	55.218	0.568	0.533
*Karenia brevis* (combined strains) SRX551359, SRX551258-61	8748	2,985,699	51	0.048	0.444	54.211	0.583	0.536
*Amphidinium carterae* CCMP1314 SRX722011	12,578	1,866,927	88	0.025	0.431	54.339	0.585	0.533
*Amphidinium massartii* SRX551299	9848	3,234,642	74	0.055	0.448	53.029	0.619	0.546
*Levanderina fissa* SRX730950-55	8601	2,852,315	93	0.107	0.477	51.881	0.664	0.571
*Crypthecodinium cohnii* SRX551367-8, SRX551296-97	6884	2,285,099	26	0.095	0.472	50.736	0.668	0.557
*Polarella glacialis* SRX554324	6600	2,028,399	41	0.112	0.481	49.930	0.690	0.573
*Symbiodinium* sp. CCMP421 SRX551158	3962	1,244,352	18	0.124	0.489	47.615	0.720	0.580
*Prorocentrum hoffmanianum* SRX722009	7764	2,511,354	39	0.112	0.480	47.879	0.731	0.589
*Scrippsiella trochoidea* CCMP3099 SRX551166-8	8303	2,785,323	33	0.107	0.478	47.182	0.733	0.588
*Alexandrium tamarense* SRX554029-30	8583	2,785,359	35	0.160	0.508	42.275	0.803	0.623
*Prorocentrum minimum* CCMP2233 SRX551159-61	1862	599,994	7	0.204	0.534	38.466	0.863	0.645
*Lingulodinium polyedrum* SRX554063-5	8701	2,854,932	53	0.210	0.536	36.406	0.884	0.659
*Alexandrium margalefi* SRX551350	7597	2,148,204	81	0.194	0.527	36.853	0.888	0.653

## References

[1] Anderson D.M. (1994). Red tides. Sci. Am..

[2] Wang D.Z. (2008). Neurotoxins from marine dinoflagellates: A brief review. Mar. Drugs.

[3] Harada T., Oshima Y., Yasumoto T. (1982). Structures of two paralytic shellfish toxins, gonyautoxins V and VI, isolated from a tropical dinoflagellate, *Pyrodinium bahamense* var. *compressa*. Agric. Biol. Chem..

[4] Baden D.G. (1989). Brevetoxins: Unique polyether dinoflagellate toxins. FASEB J..

[5] Seki T., Satake M., Mackenzie L., Kaspar H.F., Yasumoto T. (1995). Gymnodimine, a new marine toxin of unprecedented structure isolated from New Zealand oysters and the dinoflagellate, *Gymnodinium* sp. Tetrahedron Lett..

[6] Morse D., Milos P.M., Roux E., Hastings J.W. (1989). Circadian regulation of bioluminescence in *Gonyaulax* involves translational control. Proc. Natl. Acad. Sci. USA.

[7] Morey J.S., Monroe E.A., Kinney A.L., Beal M., Johnson J.G., Hitchcock G.L., Van Dolah F.M. (2011). Transcriptomic response of the red tide dinoflagellate, *Karenia brevis*, to nitrogen and phosphorus depletion and addition. BMC Genom..

[8] Lidie K.B., Ryan J.C., Barbier M., Van Dolah F.M. (2005). Gene expression in Florida red tide dinoflagellate *Karenia brevis*: Analysis of an expressed sequence tag library and development of DNA microarray. Mar. Biotechnol..

[9] Bartel D.P. (2004). MicroRNAs: Genomics, biogenesis, mechanism, and function. Cell.

[10] Proudfoot N. (2000). Connecting transcription to messenger RNA processing. Trends Biochem. Sci..

[11] Shirley B.W., Meagher R.B. (1990). A potential role for RNA turnover in the light regualtion of plant gene expression: Ribulose-1, 5-bisphosphate carboxylase small subunit in soybean. Nucleic Acids Res..

[12] Hershberg R., Petrov D.A. (2008). Selection on codon bias. Annu. Rev. Genet..

[13] Baumgarten S., Bayer T., Aranda M., Liew Y.J., Carr A., Micklem G., Voolstra C.R. (2013). Integrating microRNA and mRNA expression profiling in *Symbiodinium microadriaticum*, a dinoflagellate symbiont of reef-building corals. BMC Genom..

[14] Gao D., Qiu L., Hou Z., Zhang Q., Wu J., Gao Q., Song L. (2013). Computational Identification of MicroRNAs from the Expressed Sequence Tags of Toxic Dinoflagellate *Alexandrium tamarense*. Evol. Bioinform. Online.

[15] Morey J.S., Van Dolah F.M. (2013). Global analysis of mRNA half-lives and de novo transcription in a dinoflagellate, *Karenia brevis*. PLoS ONE.

[16] Novoa E.M., de Pouplana L.R. (2012). Speeding with control: Codon usage, tRNAs, and ribosomes. Trends Genet..

[17] Li Y.D., Li Y.Q., Chen J.S., Dong H.J., Guan W.J., Zhou H. (2006). Whole genome analysis of non-optimal codon usage in secretory signal sequences of *Streptomyces coelicolor*. Biosystems.

[18] Xu Y., Ma P., Shah P., Rokas A., Liu Y., Johnson C.H. (2013). Non-optimal codon usage is a mechanism to achieve circadian clock conditionality. Nature.

[19] Zhou M., Guo J., Cha J., Chae M., Chen S., Barral J.M., Sachs M.S., Liu Y. (2013). Non-optimal codon usage affects expression, structure and function of clock protein FRQ. Nature.

[20] Hershberg R., Petrov D.A. (2009). General rules for optimal codon choice. PLoS Genet..

[21] Peden J.F. (2000). Analysis of Codon Usage. Ph.D. Thesis.

[22] Bensoussan C., Rival N., Hanquet G., Colobert F., Reymond S., Cossy J. (2014). Isolation, structural determination and synthetic approaches toward amphidinol 3. Nat. Prod. Rep..

[23] Houdai T., Matsuoka S., Matsumori N., Murata M. (2004). Membrane-permeabilizing activities of amphidinol 3, polyene-polyhydroxy antifungal from a marine dinoflagellate. Biochim. Biophys. Acta Biomembr..

[24] Irwin B., Heck J.D., Hatfield G.W. (1995). Codon Pair Utilization Biases Influence Translational Elongation Step Times. J. Biol. Chem..

[25] Kudla G., Murray A.W., Tollervey D., Plotkin J.B. (2009). Coding-sequence determinants of gene expression in Escherichia coli. Science.

[26] Houdai T., Matsuoka S., Murata M., Satake M., Ota S., Oshima Y., Rhodes L.L. (2001). Acetate labeling patterns of dinoflagellate polyketides, amphidinols 2, 3 and 4. Tetrahedron.

[27] Kellmann R., Stüken A., Orr R.J., Svendsen H.M., Jakobsen K.S. (2010). Biosynthesis and molecular genetics of polyketides in marine dinoflagellates. Mar. Drugs.

[28] Van Wagoner R.M., Satake M., Wright J.L. (2014). Polyketide biosynthesis in dinoflagellates: What makes it different?. Nat. Prod. Rep..

[29] Bachvaroff T.R., Place A.R., Coats D.W. (2009). Expressed sequence tags from *Amoebophrya* sp. infecting *Karlodinium veneficum*: Comparing host and parasite sequences. J. Eukaryot. Microbiol..

[30] Williams E.W., Place A.R., Kim H.G., Reguera B., Hallengraeff G.M., Lee C.K., Han M.S., Choi J.K. (2014). Proliferation of 5-hydroxymethyl uracil in the genomes of dinoflagellates is synapomorphic to dinokaryon containing species. Harmful Algae, Proceedings of the 15th International Conference on Harmful Algae, Changwon, Korea, 29 October–2 November 2012.

[31] Jones G.D., Williams E.P., Place A.R., Jagus R., Bachvaroff T.R. (2015). The alveolate translation initiation factor 4E family reveals a custom toolkit for translational control in core dinoflagellates. BMC Evol. Biol..

[32] Presnyak V., Alhusaini N., Chen Y.H., Martin S., Morris N., Kline N., Olson S., Weinberg D., Baker K.E., Graveley B.R. (2015). Codon optimality is a major determinant of mRNA stability. Cell.

[33] Janouškovec J., Gavelis G.S., Burki F., Dinh D., Bachvaroff T.R., Gornik S.G., Bright K.J., Imanian B., Strom S.L., Delwiche C.F. (2017). Major transitions in dinoflagellate evolution unveiled by phylotranscriptomics. Proc. Natl. Acad. Sci. USA.

[34] Gornik S.G., Ford K.L., Mulhern T.D., Bacic A., McFadden G.I., Waller R.F. (2012). Loss of nucleosomal DNA condensation coincides with appearance of a novel nuclear protein in dinoflagellates. Curr. Biol..

[35] Talbert P.B., Henikoff S. (2012). Chromatin: Packaging without nucleosomes. Curr. Biol..

[36] Spector D.L., Triemer R.E. (1981). Chromosome structure and mitosis in the dinoflagellates: An ultrastructural approach to an evolutionary problem. Biosystems.

[37] Chatton E. (1920). Les Péridiniens Parasites: Morphologie, Reproduction, Ethologie. Arch. Zool. Exp. Gen..

[38] Bütschli O. (1880). Protozoa, Bronns Klassen und Ordnungen im Tierreich.

[39] Shoguchi E., Shinzato C., Kawashima T., Gyoja F., Mungpakdee S., Koyanagi R., Takeuchi T., Hisata K., Tanaka M., Fujiwara M. (2013). Draft assembly of the *Symbiodinium minutum* nuclear genome reveals dinoflagellate gene structure. Curr. Biol..

[40] Lin S., Cheng S., Song B., Zhong X., Lin X., Li W., Li L., Zhang Y., Zhang H., Ji Z. (2015). The *Symbiodinium kawagutii* genome illuminates dinoflagellate gene expression and coral symbiosis. Science.

[41] Aranda M., Li Y., Liew Y.J., Baumgarten S., Simakov O., Wilson M.C., Piel J., Ashoor H., Bougouffa S., Bajic V.B. (2016). Genomes of coral dinoflagellate symbionts highlight evolutionary adaptations conducive to a symbiotic lifestyle. Sci. Rep..

[42] Bachvaroff T.R., Gornik S.G., Concepcion G.T., Waller R.F., Mendez G.S., Lippmeier J.C., Delwiche C.F. (2014). Dinoflagellate phylogeny revisited: Using ribosomal proteins to resolve deep branching dinoflagellate clades. Mol. Phylogenet. Evol..

[43] Altschul S.F., Gish W., Miller W., Myers E.W., Lipman D.J. (1990). Basic local alignment search tool. J. Mol. Biol..

